# A Realist Inquiry Exploring a Ward Based Physical Activity Service in a Psychiatric Intensive Care Unit

**DOI:** 10.1192/bjo.2025.10094

**Published:** 2025-06-20

**Authors:** Toby Keel, Mehtab Ghazi Rahman, Matt Waugh

**Affiliations:** ^1^Loughborough University, Loughborough, United Kingdom; ^2^Central & North West London NHS Foundation Trust, London, United Kingdom

## Abstract

**Aims:** This realist evaluation explored the implementation and effectiveness of a novel physical activity (PA) service within a male Psychiatric Intensive Care Unit (PICU) at St Charles Hospital, London. The study aimed to understand “what works, for whom, how, and under what circumstances” regarding the PA service’s impact on patient well-being and staff experiences. The evaluation aimed to develop and refine programme theories explaining the relationships between the PICU’s resources, context, and the mechanisms generating observed outcomes, ultimately informing recommendations for future PICU PA programme design.

**Methods:** A realist inquiry framework, utilizing the Context-Intervention-Actor-Response Mechanism-Outcome Configuration (CIAMOc), guided the evaluation. Data collection, aligned with RAMESIS II reporting standards, spanned six months (March–October 2023) and employed a mixed-methods approach. This included 85 hours of ethnographic observation on the ward, semi-structured interviews with 18 staff and 9 patients, and two staff focus groups. Data analysis involved iterative coding and theory refinement using a CIAMO heuristic tool. Candidate programme theories, derived from a prior realist synthesis, were deductively explored and inductively refined through the three phases of realist interviewing (theory gleaning, refinement, and consolidation).

**Results:** Eighteen interlinked programme theories were developed, highlighting the critical role of leadership, the physical environment, and the social environment in PA service success. Findings emphasised that stable leadership, staff retention, and a cohesive team were foundational for creating a therapeutic culture conducive to PA implementation. Leadership “buy-in”, demonstrated through valuing and supporting PA, empowered staff and facilitated efficient processes. Redeveloping the ward gym with safe, tailored equipment and maximizing access to diverse PA opportunities (gardening, sports) promoted patient agency and engagement. The dedicated Physical Activity Nurse (PAN) played a crucial role in motivating patients, fostering positive social interactions, and normalizing PA experiences. Preliminary findings suggest that the PA service contributed to reduced violent incidents, weight gain, and improved staff job satisfaction, though further analysis is exploring the specific mechanisms driving these outcomes.

**Conclusion:** This realist evaluation provides valuable insights into the complex factors influencing the success of PA programmes in PICU settings. The study demonstrates that a multi-component approach, addressing leadership, physical environment, and social dynamics, is essential for effective implementation. By understanding the underlying mechanisms and contextual influences, this research can inform the development of more effective and sustainable PA interventions that promote patient well-being, reduce restrictive practices, and enhance staff experiences within PICUs. Further analysis will explore the specific mechanisms linking PA to positive outcomes, providing a stronger evidence base for future programme design and implementation.

